# Equal Partners, Prioritized Lives? Preschoolers' Evaluative Patterns Toward Humans and Robots in Conventional and Moral Domains

**DOI:** 10.1002/pchj.70122

**Published:** 2026-09-03

**Authors:** Xiao Zeng, Zixin Wu, Junjing Feng, Zhiqiang Yan, Hao Tan

**Affiliations:** ^1^ School of Education, Hunan First Normal University Changsha China; ^2^ Department of Psychology Hunan Normal University Changsha China; ^3^ Cognition and Human Behavior Key Laboratory of Hunan Province, Hunan Normal University Changsha China; ^4^ School of Intelligent Manufacturing, Hunan First Normal University Changsha China

**Keywords:** convention, moral, preschoolers, robot, social domain theory

## Abstract

As social robots become increasingly integrated into children's daily lives, it remains unclear whether children apply the same normative standards to artificial agents as they do to humans. Drawing on social domain theory and a dual‐process‐informed distinction between categorical choice and graded appraisal, the present research examined preschoolers' binary decisions and appropriateness ratings concerning humans and robots in the conventional domain (Study 1) and the moral domain (Study 2). Two studies used a modified road‐accident dilemma task. Across both studies, children's choices were strongly shaped by the behavioral valence of the sacrificial and saved targets; repeated‐measures ANOVAs also suggested greater intervention in human than robot scenarios, although these agent‐related choice effects were model‐sensitive in LMM checks. Appropriateness ratings showed a domain‐related pattern. In Study 1, ratings were comparable in human and robot scenarios, consistent with an egalitarian application of conventional standards. In Study 2, children rated saving an immoral human as more appropriate than saving an immoral robot, whereas ratings did not differ when the saved agents were moral. Because the direct cross‐study interaction did not reach the conventional significance threshold, the domain contrast should be treated as suggestive rather than conclusive. These findings suggest that preschoolers may treat robots as procedural social actors in conventional contexts while granting humans greater presumed moral standing in morally consequential contexts.

## 1 Introduction

A fundamental premise in social psychology is that observable behavior constitutes the primary basis upon which individuals evaluate others (Reeder 2009). The adage “actions speak louder than words” encapsulates the widespread human tendency to infer character, intentions, and trustworthiness from others' prosocial or antisocial conduct. With rapid advances in artificial intelligence, social robots are becoming increasingly present in children's everyday environments, positioning them as novel social actors alongside human agents (Zhou et al. 2024, 2023). Research informed by the Computers Are Social Actors (CASA) framework demonstrates that people often spontaneously apply human‐like social expectations, norms, and evaluative standards to artificial agents (Gambino et al. 2020; Nass and Moon 2000). This tendency may be especially relevant in early childhood, a developmental period characterized by animistic thinking—the attribution of life, intention, and agency to non‐living entities (Looft and Bartz 1969; Severson and Lemm 2016). Given that young children rely heavily on behavioral cues when evaluating human agents, a critical question arises as to whether children extend this behavior‐based evaluative mechanism to robots, or whether agent type functions as a boundary condition that shapes how observed actions are interpreted and weighted.

Addressing this question requires recognizing that children's evaluations of social norm violations are not unitary but organized into distinct domains. Accordingly, the influence of agent type may vary depending on the domain in which a behavior occurs. According to social domain theory (Glassman and Zan 1995; Smetana 2013; Turiel 1983), children distinguish between conventional and moral domains from an early age. Conventional norms (e.g., queuing, etiquette) are socially constructed, consensus‐based rules designed to coordinate social order and facilitate smooth interpersonal interaction. In contrast, moral norms (e.g., prohibitions against harm) concern issues of intrinsic welfare, justice, and rights and are understood as relatively independent of social consensus or authority (Heath 2017). This domain distinction offers a critical theoretical lens for research on human–robot interaction. Conventions function as procedural scripts that regulate social coordination and, in principle, can be followed by any social actor; accordingly, children may expect robots to comply with conventional norms in ways similar to humans. Moral norms, however, are more closely tied to assumptions about sentience, vulnerability, and the capacity to experience harm (Perry 2024; Pringle et al. 2024). As a result, children's judgments of humans and robots may diverge across domains: children may apply conventional rules similarly to both agents, while granting humans stronger moral priority when intrinsic welfare is implicated.

### 1.1 Moral Judgments in the Conventional Domain

According to social domain theory, conventions constitute a conceptual system that is distinct from moral imperatives. Conventions are defined as socially agreed‐upon, context‐dependent behavioral rules that are established and maintained through social consensus and authority, such as norms governing etiquette, dress codes, and public order (Hardecker et al. 2016; Heath 2017). Unlike moral norms, which are grounded in intrinsic concerns such as welfare, fairness, and justice, conventional norms primarily serve to coordinate social interactions, regulate collective behavior, and signal group membership. Consistent with this distinction, research shows that conventional transgressions are typically judged as less serious than moral violations and are viewed as more contingent upon explicit rules or authoritative endorsement (de Oliveira et al. 2020; Tisak and Turiel 1988). Nevertheless, despite their relative flexibility compared to moral principles, conventions play a crucial role in maintaining predictability and preserving the structural coherence of everyday social exchanges.

From a developmental perspective, the acquisition and enforcement of conventional norms are central components of early socialization. Empirical studies indicate that preschool‐aged children are highly attuned to normative regularities and often display pronounced rule rigidity or rule diligence within the conventional domain (Hardecker et al. 2016; Schmidt and Rakoczy 2023). During this developmental period, children place strong emphasis on the faithful execution of established social scripts as a means of sustaining social order (Ma 2013). Rules, once introduced by an authority figure or stabilized through repeated practice, are perceived as binding within the immediate context. Consequently, when evaluating conventional behaviors, young children tend to prioritize whether an action conforms to the rule itself rather than characteristics of the actor who performs it (Rakoczy et al. 2008; Turiel 2008). This focus on observable behavioral compliance implies that disruptions to social order (e.g., cutting in line) are salient violations for young children, even when the actor's intentions or social identity are not central to the judgment.

This developmental emphasis on rule‐based evaluation raises important questions for human–robot interaction. Drawing on the CASA paradigm (Nass and Moon 2000), prior research suggests that individuals often apply human social scripts and expectations to artificial agents in an automatic manner (Eng et al. 2023; Zhou et al. 2023). CASA and social domain theory address complementary aspects of children's judgments of robots: CASA concerns whether a technological entity is treated as a socially relevant participant, whereas social domain theory specifies the criteria children use to evaluate conduct within a given normative domain. Once a robot is treated as a participant, conventional judgments may depend primarily on observable compliance with context‐specific coordination rules. Because such rules are consensus‐based procedures for organizing social interaction, children may apply them in a relatively agent‐general manner, treating ontological status as secondary to rule adherence. Under this account, robots may be evaluated as functional social partners in conventional contexts, without implying that they are granted the same moral standing as humans. This relatively agent‐general application of conventional rules may be further amplified in early childhood due to children's propensity for animistic thinking and their readiness to attribute agency to non‐human entities (Looft and Bartz 1969; Merewether 2018). As a result, young children may perceive robots as potential participants in the social order governed by conventional norms. If conventional judgments are primarily driven by script‐based processing—where compliance with established procedures constitutes the central evaluative criterion—children should extend similar normative expectations to human and robotic agents. Accordingly, in contexts involving procedural rules such as queuing (Surian and Margoni 2020), children may enforce conventional rules in a similar manner, evaluating human and robot compliance or violation according to equivalent standards, with the agent's ontological status becoming secondary to the preservation of the rule itself (Ayalon et al. 2023).

### 1.2 Moral Judgments in the Moral Domain

In contrast to the conventional domain, the moral domain concerns issues of intrinsic welfare, justice, and rights. According to social domain theory, moral norms such as prohibitions against physical harm or obligations to act fairly are understood as relatively independent of social consensus, cultural convention, or the dictates of authority figures (Smetana and Yoo 2023; Turiel 2008). Rather, their prescriptive force derives from the inherent consequences of actions for the well‐being of others, particularly victims who may be harmed or wronged. Extensive research has demonstrated that moral principles are often treated as broadly applicable and obligatory, such that acts that cause harm are judged as wrong regardless of whether explicit rules exist to prohibit them (Cushman 2015; Giffin and Lombrozo 2017). Consequently, unlike the procedural and context‐sensitive nature of conventional norms, the moral domain is fundamentally anchored in the protection of rights and the prevention of suffering, with evaluative focus placed on outcomes and their impact on the victim.

From a developmental perspective, children's moral cognition is closely linked to their emerging capacity for empathy and their understanding of biological vulnerability (Zhang et al. 2025; Zhou et al. 2024). Empirical evidence suggests that even infants exhibit rudimentary moral sensitivities, systematically preferring agents who help others over those who hinder them (Tan and Hamlin 2024; Van de Vondervoort and Hamlin 2017). As children develop, their moral evaluations become increasingly tied to the concept of sentience (Perry 2024), defined as the capacity to experience pain, emotions, and subjective states. Developmental research indicates that children often reason about biological properties in essentialist ways: they recognize that humans possess internal biological states and a capacity for suffering (Zhang et al. 2025), which together ground moral entitlement and protection (Van de Vondervoort and Hamlin 2016). Drawing on psychological essentialism, children may distinguish natural kinds (e.g., humans) from artifacts (e.g., robots) based on perceived internal properties or essences, and may attribute biological reality and subjective vulnerability more readily to natural kinds than to artifacts (Gelman 2003; Keil 1989). Accordingly, when children evaluate moral transgressions, they do not simply register rule violations; rather, they consider the victim's capacity to be harmed. This sensitivity to victimhood suggests that moral judgments are shaped by children's representations of the biological and psychological reality of the entities involved.

This essentialist grounding of morality introduces a critical boundary condition for human–robot interaction. Although the CASA framework suggests that children may interact with robots using social heuristics (Nass and Moon 2000), the moral domain may foreground an ontological distinction between biological beings and artifacts. Although children may attribute agency, intention, or even social roles to robots, they may still regard humans as possessing richer biological and experiential capacities (Perry 2024; Zhou et al. 2024). This distinction gives rise to an anthropocentric bias, particularly in morally consequential contexts involving protection, harm, or sacrifice (Reynolds and Ceranic 2007; Sommer et al. 2019). Within the present framework, these contrasting patterns are described as an egalitarian pattern—where conventional standards are applied equivalently across agent types (Ayalon et al. 2023)—and an anthropocentric pattern—where humans are prioritized in morally consequential evaluations (Wilks et al. 2020). Rather than reflecting a reflective, conscious theory of ontological status, these patterns are hypothesized to arise from domain‐specific cognitive processes and behavioral heuristics (Smetana 2013; Turiel 2008), such that children may prioritize human welfare over that of robots and may construe the protection of human life as carrying greater moral weight than the protection of artificial agents.

Despite these theoretical insights, an important empirical gap remains: how young children evaluate human versus robotic agents across distinct normative domains has rarely been systematically examined within a unified comparative framework. Prior work in human–robot interaction has typically investigated children's conventional rule understanding or moral reasoning in isolation, leaving it unclear whether preschoolers apply uniform evaluative principles to artificial agents or exhibit domain‐differentiated evaluative patterns. Moreover, previous developmental research has seldom examined whether children's social evaluations of artificial agents diverge when expressed through categorical behavioral choices versus reflective normative appraisals. Addressing these unresolved gaps is critical for delineating the boundary conditions of children's social categorization of non‐human entities and clarifying how domain‐specific cognitive mechanisms guide human–robot interaction.

### 1.3 Current Studies

Across both studies, we employed a modified road‐accident dilemma paradigm with parallel numerical, intervention, and rating structures (Zhou et al. 2024). Study 1 targets the conventional domain, focusing on children's evaluations of adherence to arbitrary rules that structure social order. Study 2 targets the moral domain, examining judgments in situations involving intrinsic welfare, harm, and moral obligation. Across both studies, agent type (human vs. robot) and behavioral valence (compliant/moral vs. transgressive/immoral) were systematically manipulated to determine whether children apply a relatively unified evaluative standard to human and robotic agents or whether their judgments are differentiated as a function of the agent's type.

Drawing on a dual‐process‐informed distinction between categorical action selection and graded explicit appraisal (Cushman 2013; Greene 2007), we propose that children's binary choices and subsequent appropriateness ratings may weight the available information differently. The binary task requires a categorical selection between maintaining and diverting the vehicle's path, whereas the rating task allows a graded normative appraisal of the selected action. Rather than assuming that these measures provide process‐pure assessments of separate cognitive systems, we treat them as functionally distinct response formats that may encourage different weighting of target valence, agent type, and normative domain. Specifically, we developed the following hypotheses:

*In conventional contexts, children's binary choices are expected to be sensitive to agents' prior rule‐related behavior and to show a general tendency toward greater intervention in human than in robot scenarios*.

*In conventional contexts, children's appropriateness ratings are expected to be guided primarily by rule‐based conventional scripts, producing comparable ratings in human and robot scenarios after agents' prior rule‐related behavior is taken into account*.

*In moral contexts, children's binary choices are expected to be sensitive to agents' prior moral behavior and to show a general tendency toward greater intervention in human than in robot scenarios*.

*In moral contexts, children's appropriateness ratings are expected to show a conditional anthropocentric pattern: saving an immoral human will be rated as more appropriate than saving an immoral robot, whereas ratings for saving moral humans and moral robots will be comparable*.

## 2 Study 1: Preschoolers' Judgments of Humans and Robots in the Conventional Domain

### 2.1 Participants

An a priori power analysis using G*Power (version 3.1.9.7) indicated that a minimum of 36 participants was required to achieve a statistical power of 0.95 (*f* = 0.25, *α* = 0.05) for the repeated‐measures design (Faul et al. 2009). A final sample of 36 typically developing preschoolers (21 boys, 15 girls; *M*_age_ = 5.71 years, SD = 0.45) was recruited from a public kindergarten in Changsha, China. None had a reported history of language or social impairments. The study protocol was approved by the Institutional Review Board of Hunan Normal University. Written informed consent was obtained from parents, and verbal assent was provided by each child.

### 2.2 Design

Study 1 employed a 2 (Agent Type: Human vs. Robot) × 2 (Sacrificial Target Behavior: Compliant vs. Transgressive) × 2 (Saved Target Behavior: Compliant vs. Transgressive) within‐subjects factorial design. Using a modified road‐accident dilemma paradigm, the study assessed two dependent measures: children's binary intervention choices and their appropriateness ratings of the selected action.

### 2.3 Materials and Measures

#### 2.3.1 Character Induction

Following previous research (Van de Vondervoort and Hamlin 2017), visual stimuli depicted human and robotic agents color‐coded by behavioral valence to facilitate discrimination. For example, in one counterbalancing version, compliant agents who followed social rules wore blue or white, whereas transgressive agents who violated social rules wore red. Crucially, the assignment of colors to behavioral valence was fully counterbalanced across participants to control for potential color biases.

At the beginning of the session, the experimenter verified children's understanding of conventional norms. Age‐appropriate definitions were provided, describing compliant behaviors as adhering to social rules (e.g., waiting for one's turn, keeping public areas tidy) and transgressive behaviors as violating social order (e.g., cutting in line, making a mess). Children were then trained on a 4‐point visual scale represented by circles of increasing size, ranging from “very inappropriate” (smallest circle) to “very appropriate” (largest circle). To ensure comprehension of the scale's ordinal nature, children were asked to compare intermediate values, and only those who demonstrated clear understanding proceeded.

Following this, a character induction task was administered using three everyday social scenarios (waiting in line, offering a seat, and disposing of trash) to establish the agents' conventional valence. The experimenter narrated a randomly selected scenario (e.g., a compliant agent queuing properly vs. a transgressive agent cutting in line) and asked the child to retell the events. Children then rated each agent's behavior using the 4‐point scale. This served as a manipulation check; only children who accurately identified the actions and correctly differentiated the agents' behavioral valence advanced to the main study.

#### 2.3.2 Road‐Accident Dilemma Task

**FIGURE 1:**
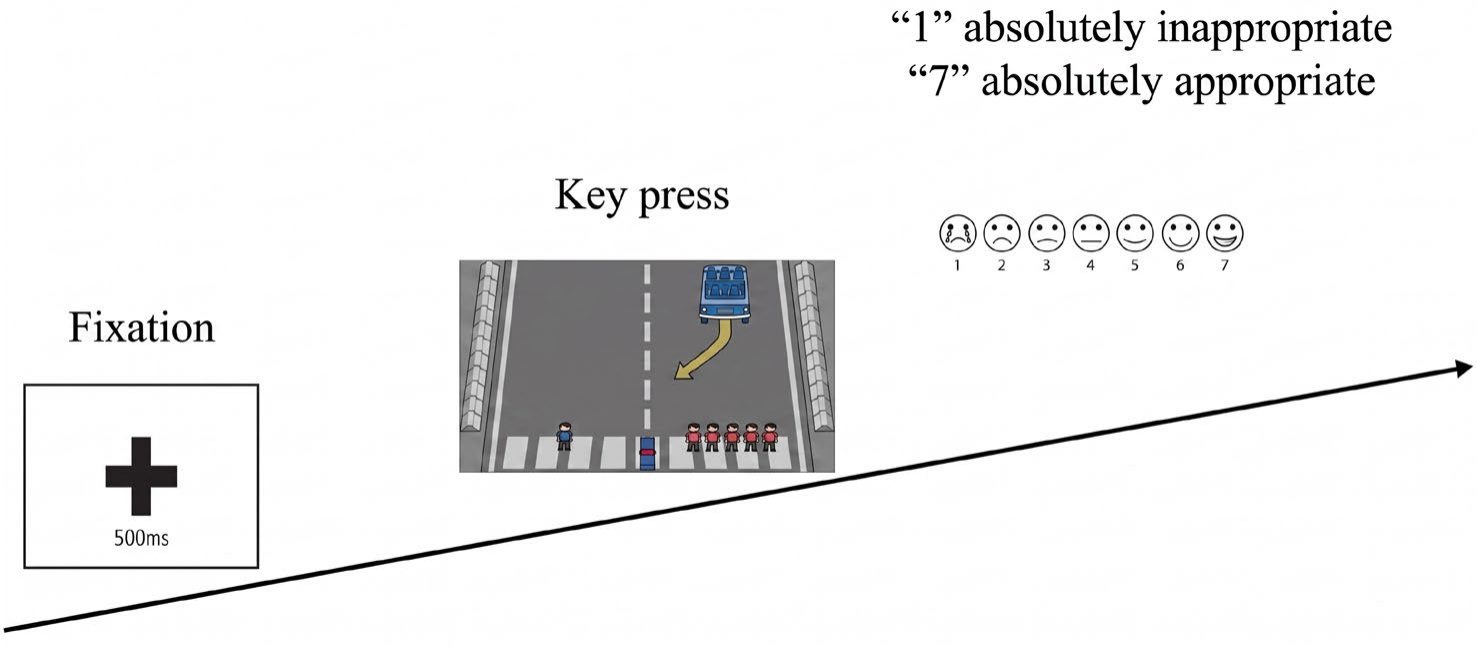
Study 1 flowchart.

Children's intervention choices and appropriateness judgments were assessed using a modified road‐accident dilemma task (Mayer et al. 2022; Zhou et al. 2024), See Figure 1. First, participants were trained on a 7‐point appropriateness scale, which used facial expressions ranging from “very unhappy” (1 = completely inappropriate) to “very happy” (7 = completely appropriate) (Liu et al. 2025). Practice trials were conducted to verify that children understood the ordinal nature of the scale end‐points and intermediate values.

The formal study consisted of 32 trials divided into eight blocks, with four trials in each block. These blocks were defined by crossing agent type, which included human and robot agents, and scenario type, which reflected norm compliance and group composition. The eight blocks included the following conditions: (1) Two blocks presented symmetrical one‐versus‐one scenarios with human agents sharing the same behavioral status, such as one transgressive human facing another transgressive human, or one compliant human facing another compliant human; (2) Two blocks mirrored this setup with robot agents, for example, one transgressive robot against another transgressive robot, or one compliant robot against another compliant robot; (3) Two blocks introduced numerical conflict scenarios involving human agents. In these, a group of five agents with one behavioral status was contrasted with a single agent exhibiting the opposite status, such as five compliant humans versus one transgressive human; (4) The final two blocks presented similar numerical conflict scenarios with robot agents, for example, five transgressive robots versus one compliant robot. These blocks allowed for systematic examination of how children balance conventional compliance and numerical outcome considerations when making decisions about human and robotic agents.

To control for order and spatial effects, the presentation order of blocks was randomized across participants, and the spatial position of agents (left/right roads), the numerical configuration (1 vs. 5 on the main road), and the vehicle's direction were fully counter‐balanced. In each trial, children viewed a dilemma image, such as a vehicle approaching a group of transgressive robots on the main road while a single compliant robot stood on the side road, and were asked to make a binary decision about whether to press the switch to turn the vehicle. Immediately following their choice, children rated the appropriateness of their decision using the 7‐point smiley face scale, where higher scores indicated a stronger belief that the chosen action was appropriate.

### 2.4 Procedure

Data collection was conducted individually in a quiet room at the kindergarten, with sessions lasting approximately 10 min. The procedure comprised two consecutive phases. The session began with the character induction phase. Children were introduced to the agent types (humans vs. robots) and briefed on the relevant normative concepts (conventional rules). After completing the scale training, the narrative induction task was administered. This served as a strict inclusion criterion: only participants who successfully differentiated between compliant and transgressive agents proceeded to the formal study.

Next, participants completed the road‐accident dilemma task. Prior to the formal trials, they received training on using the 7‐point appropriateness scale. Following this, the 32 test trials were presented in randomized blocks. In each trial, children followed a two‐step response process: first, they made a binary decision via keyboard to maintain or divert the vehicle's path; immediately thereafter, they rated the appropriateness of their decision on the 7‐point scale. The task was self‐paced, allowing unlimited time for response. Upon completion, each child received a small gift as a token of appreciation.

### 2.5 Analytic Plan

Data analyses were performed using JASP (0.95.4) and R (4.6.0) for sensitivity checks. To address the primary research questions, a repeated‐measures ANOVA was performed. For each condition, two dependent variables were derived. First, the proportion of intervention choices was calculated as the ratio of intervention choices to the total number of trials within that condition. Second, the mean appropriateness score was obtained by averaging participants' ratings regarding the appropriateness of their decisions. Because binary choice proportions are bounded and may show limited variance in dilemma paradigms, random‐intercept linear mixed‐effects models (LMMs) were also conducted as sensitivity analyses to evaluate the robustness of the ANOVA‐based choice results under alternative error assumptions.

### 2.6 Results

To examine decision‐making patterns, a 2 (agent type) × 2 (sacrificial target valence) × 2 (saved target valence) repeated‐measures ANOVA was conducted on the proportion of intervention choices. The analysis yielded significant main effects of agent type [*F*(1, 35) = 6.76, *p* = 0.014, $\eta_{P}^{2}$ = 0.16], sacrificial target valence [*F*(1, 35) = 20.57, *p* < 0.001, $\eta_{P}^{2}$ = 0.37], and saved target valence [*F*(1, 35) = 480.11, *p* < 0.001, $\eta_{P}^{2}$ = 0.93]. Overall, children were more likely to divert the vehicle in human scenarios than in robot scenarios. They also showed a higher tendency to intervene when the sacrificial target was transgressive and when the agents to be saved were compliant. A significant interaction emerged between sacrificial target behavior and saved target behavior [*F*(1, 35) = 15.75, *p* < 0.001, $\eta_{P}^{2}$ = 0.31]. Bonferroni‐corrected post hoc comparisons revealed a ceiling effect when the saved target was compliant, where the proportion of intervention choices was universally high regardless of whether the sacrificial agent was compliant or transgressive. In contrast, when the saved target was transgressive, decisions were highly sensitive to the sacrificial agent's character. Children were significantly more likely to sacrifice the agent if that agent was transgressive than if the agent was compliant. No other significant interactions were observed (*p*s > 0.05). To evaluate robustness under alternative error assumptions, we fitted a random‐intercept linear mixed‐effects model (LMM) on the choice proportions. The LMM reproduced the strong target‐behavior effects, all *p*s < 0.001, but it did not reproduce the ANOVA‐based main effect of agent type. Thus, the agent‐type effect in children's choices should be interpreted as model‐sensitive (Table 1).

**TABLE 1:** Descriptive statistics of Study 1 (*M*/SD).

Agent type	Sacrificial target	Saved target	Dependent variable	
			Proportion of intervention choices	Appropriateness rating
Human	Compliant	Compliant	0.99/0.06	5.76/1.34
		Transgressive	0.03/0.10	1.94/0.91
	Transgressive	Compliant	0.99/0.04	6.20/1.16
		Transgressive	0.32/0.41	2.84/1.79
Robot	Compliant	Compliant	0.97/0.14	5.85/1.45
		Transgressive	0.01/0.06	1.81/0.98
	Transgressive	Compliant	0.99/0.04	6.13/1.07
		Transgressive	0.26/0.38	2.65/1.68

A repeated‐measures ANOVA was conducted on the appropriateness ratings. The analysis revealed a significant main effect of the sacrificial target valence [*F*(1, 35) = 14.19, *p* < 0.001, $\eta_{P}^{2}$ = 0.29]. Participants rated their intervention choices as significantly more appropriate when the sacrificial target was transgressive compared to when the target was compliant. Additionally, a significant main effect of the saved target valence was found [*F*(1, 35) = 168.96, *p* < 0.001, $\eta_{P}^{2}$ = 0.83]. Children rated decisions that resulted in saving compliant agents as significantly more appropriate than those saving transgressive agents. However, the main effect of agent type was not significant [*F*(1,35) = 0.86, *p* = 0.360, $\eta_{P}^{2}$ = 0.02], indicating that children's evaluations did not differ between human and robot scenarios. Furthermore, no significant interaction effects were observed among the three variables (*p*s > 0.05), suggesting that children's explicit evaluations were primarily organized by the prior conventional behavior of the sacrificial and saved targets rather than by agent type.

### 2.7 Discussion

Study 1 partly supported Hypothesis 1. Children's intervention choices and appropriateness ratings were strongly organized by agents' prior conventional behavior: children were more likely to intervene when the sacrificial target was transgressive and when the agents to be saved were compliant. The repeated‐measures ANOVA also suggested a greater tendency to intervene in human than in robot scenarios. However, this agent‐type effect in choices was not reproduced in the LMM sensitivity analysis and should therefore be interpreted as model‐sensitive. In contrast, appropriateness ratings revealed no significant difference between human and robot scenarios, supporting the hypothesis that children's explicit evaluations in the conventional domain would be guided primarily by rule‐based conventional scripts.

The observation that children's explicit normative evaluations prioritize observable rule compliance over agent type aligns with the CASA framework (Nass and Moon 2000) and developmental theories of animism (Looft and Bartz 1969). Preschoolers characterized by preoperational thinking tend to attribute agency to interactive entities regardless of biological status. Within the conventional domain, norms function as structural scripts designed to maintain social order. Evidence suggests that young children often process these norms through a lens of rule rigidity, focusing on the external execution of the rule rather than the internal nature of the actor (Brandl et al. 2015; Turiel 2024). Consequently, once a robot is perceived as an agent capable of following instructions, its violation of a convention disrupts the social script just as visibly as a human violation. The data suggest that for social conventions, children prioritize behavioral compliance over agent type and evaluate human and robot agents in relatively similar ways in conventional contexts.

However, social conventions represent only one dimension of normative evaluation specifically regarding rules governed by arbitrary consensus. It remains to be determined whether the observed equivalence between humans and robots extends to the moral domain. According to social domain theory (Smetana 2013; Turiel 1983, 2008, 2010), moral judgments differ fundamentally from conventions as they are grounded in intrinsic welfare and the prevention of harm. Unlike procedural rules, moral obligations are typically tied to the biological reality of the victim. Study 2 therefore examined whether children's decisions and appropriateness ratings would show greater differentiation between humans and robots in high‐stakes moral contexts involving welfare and harm.

## 3 Study 2: Preschoolers' Judgments of Humans and Robots in the Moral Domain

### 3.1 Participants

An a priori power analysis using G*Power (version 3.1.9.7, Faul et al. 2009) indicated that a minimum of 36 participants was required to achieve a statistical power of 0.95 (*f* = 0.25, *α* = 0.05) for the repeated‐measures design. To allow for potential attrition, 38 typically developing preschoolers (21 boys, 17 girls; *M*_age_ = 5.90 years, SD = 0.37) were recruited from a public kindergarten in Changsha, China. None had a reported history of language or social impairments. The study protocol was approved by the institutional review board of our university. Written informed consent was obtained from parents, and verbal assent was provided by each child. None of the children had participated in Study 1.

### 3.2 Design

Study 2 employed a 2 (Agent Type: Human vs. Robot) × 2 (Sacrificial Target Behavior: Moral vs. Immoral) × 2 (Saved Target Behavior: Moral vs. Immoral) within‐subjects factorial design. Consistent with Study 1, a modified road‐accident dilemma paradigm was used; however, the focal attribute shifted from conventional norms to moral norms. The study assessed two dependent measures: children's binary intervention choices and their appropriateness ratings of the selected action.

### 3.3 Materials and Task

#### 3.3.1 Character Induction

Similar to Study 1, visual stimuli depicted human and robotic agents color‐coded by behavioral valence to facilitate discrimination. For example, in one counterbalancing version, moral agents wore blue or white, whereas immoral agents wore red. Crucially, the assignment of colors to behavioral valence was fully counterbalanced across participants to control for potential color biases.

At the beginning of the session, the experimenter verified children's understanding of moral concepts. Age‐appropriate definitions were provided, describing moral behaviors as prosocial actions (e.g., protecting others, ensuring safety) and immoral behaviors as antisocial violations (e.g., physically harming others). Children were then trained on a 4‐point visual scale represented by circles of increasing size, ranging from “very immoral” (smallest circle) to “very moral” (largest circle), ensuring they understood its ordinal nature.

Following this, participants completed a character induction task that consisted of three scenarios involving dangerous driving, physical assault, and throwing objects. The experimenter narrated a randomly selected scenario (e.g., a moral agent preventing harm vs. an immoral agent throwing objects to injure others) and asked the child to retell the events. Children then rated each agent's behavior using the 4‐point scale. This served as a strict manipulation check: only children who accurately identified the actions and correctly differentiated the agents' behavioral valence advanced to the main study.

#### 3.3.2 Road‐Accident Dilemma Task

The design and administration of the moral dilemma task were identical to Study 1. The formal test phase consisted of 32 trials organized into eight blocks, structurally mirroring Study 1, but with moral valence (moral vs. immoral) replacing conventional valence as the defining attribute for the sacrificial and saved agents.

### 3.4 Procedure

The experimental procedure was identical to that of Study 1, with the exception that the familiarization and character induction phases focused on moral valence. The administration of the road‐accident dilemma task and the recording of responses followed the exact same protocol.

### 3.5 Analytic Plan

Data analysis followed the same strategy as Study 1. Repeated‐measures ANOVAs were conducted for both behavioral and rating data, with moral valence as the within‐subject factor for the sacrificial and saved targets. Because binary choice proportions are bounded and may show limited variance in dilemma paradigms, random‐intercept LMMs were also conducted as sensitivity analyses to evaluate the robustness of the ANOVA‐based choice results under alternative error assumptions.

### 3.6 Results

To examine the impact of moral adherence on decision‐making, a 2 (agent type) × 2 (sacrificial target valence) × 2 (saved target valence) repeated‐measures ANOVA was conducted on the proportion of intervention choices. The analysis revealed significant main effects for agent type [*F*(1,37) = 9.49, *p* = 0.004, $\eta_{P}^{2}$ = 0.20], sacrificial target valence [*F*(1,37) = 18.93, *p* < 0.001, $\eta_{P}^{2}$ = 0.34], and saved target valence [*F*(1,37) = 900.21, *p* < 0.001, $\eta_{P}^{2}$ = 0.96]. In the ANOVA, children were more likely to intervene in human than in robot scenarios. They were also more likely to intervene when the sacrificial target was immoral and when the agents to be saved were moral. All two‐way interactions were significant (*p*s < 0.05). More importantly, a significant three‐way interaction emerged among agent type, sacrificial target, and saved target [*F*(1,37) = 9.49, *p* = 0.004, $\eta_{P}^{2}$ = 0.20]. Bonferroni‐corrected post hoc comparisons indicated that target behavior was the dominant driver of children's choices, whereas agent type modulated responses in specific contexts. When both the sacrificial and saved targets were moral, children's choices did not differ between human and robot scenarios, *p* = 0.80, reflecting a strong tendency to save the moral group in both agent conditions. In contrast, in conditions involving immoral agents, children were more likely to intervene in human than in robot scenarios, *p*s < 0.05. This suggests that while moral adherence is the primary criterion, children's choices were primarily organized by prior moral behavior, with agent type contributing in some morally negative contexts. To assess robustness under alternative error assumptions, we fitted a random‐intercept LMM on the Study 2 proportions. The LMM reproduced the strong target‐valence effects but did not reproduce the agent main effect or the agent‐related interactions. Thus, the agent‐related choice effects in Study 2 should be interpreted as ANOVA‐based and model‐sensitive rather than as fully robust across model specifications (Table 2).

**TABLE 2:** Descriptive statistics of Study 2 (*M*/SD).

Agent type	Sacrificial target	Saved target	Dependent variable	
			Proportion of intervention choices	Moral appropriateness rating
Human	Moral	Moral	0.98/0.09	5.80/1.33
		Immoral	0.01/0.01	1.39/0.49
	Immoral	Moral	1.00/0.01	6.59/0.55
		Immoral	0.27/0.38	2.78/2.04
Robot	Moral	Moral	0.97/0.14	5.92/1.38
		Immoral	0.01/0.01	1.26/0.35
	Immoral	Moral	1.00/0.01	6.72/0.41
		Immoral	0.18/0.35	2.28/1.94

A repeated‐measures ANOVA was conducted on the moral appropriateness ratings. The analysis yielded significant main effects for sacrificial target valence [*F*(1,37) = 37.50, *p* < 0.001, $\eta_{P}^{2}$ = 0.50] and saved target valence [*F*(1,37) = 534.17, *p* < 0.001, $\eta_{P}^{2}$ = 0.94]. Consistently, children rated decisions as significantly more morally appropriate when they resulted in sacrificing immoral agents or saving moral ones. Notably, a significant interaction emerged between agent type and saved target valence [*F*(1,37) = 16.41, *p* < 0.001, $\eta_{P}^{2}$ = 0.31]. Bonferroni‐corrected post hoc comparisons revealed that when the saved target was moral, children's moral evaluations did not differ between saving a human and a robot (*p* = 0.58). However, when the saved target was immoral, participants rated saving a human as significantly more morally appropriate than saving a robot (*p* = 0.006). The main effect of agent type and other interaction effects were not significant (*p*s > 0.05). Within Study 2, this pattern indicates that anthropocentric evaluation emerged conditionally when the agents to be saved had previously behaved immorally.

To determine whether children's judgment patterns differed between conventional contexts (Study 1) and moral contexts (Study 2), a mixed‐model ANOVA was conducted with domain type (convention vs. moral) as a between‐subjects factor. Regarding the proportion of intervention choices, the analysis revealed no significant main effect of domain type, nor were there any significant interaction effects involving domain type (*p*s > 0.05). This indicates that the cross‐domain analysis did not provide evidence that children's intervention‐choice patterns differed significantly between the conventional and moral domains. In contrast, the analysis of moral appropriateness scores revealed subtle contextual differences. The rating analysis produced two effects involving domain that approached but did not reach the conventional alpha level: saved target and domain type [*F*(1,72) = 3.81, *p* = 0.055, $\eta_{P}^{2}$ = 0.05], and three‐way interaction among agent type, saved target, and domain type [*F*(1,72) = 3.11, *p* = 0.082, $\eta_{P}^{2}$ = 0.041]. These effects were directionally consistent with a domain‐related difference in anthropocentrism, but neither reached the conventional threshold for statistical significance; therefore, they do not provide conclusive evidence for a cross‐domain interaction.

### 3.7 Discussion

Study 2 partly supported Hypothesis 2. Children's choices were strongly organized by agents' prior moral behavior: they were more likely to intervene when the sacrificial target was immoral and when the agents to be saved were moral. The repeated‐measures ANOVA also suggested greater intervention in human than in robot scenarios, particularly in conditions involving immoral agents. However, because the LMM sensitivity analysis did not reproduce the agent main effect or the agent‐related interactions, these choice effects should be interpreted as model‐sensitive. The more robust evidence for Hypothesis 2 came from children's moral appropriateness ratings. Saving moral humans and moral robots received comparable ratings, whereas saving an immoral human was rated as more appropriate than saving an immoral robot. Thus, children's explicit evaluations showed a conditional anthropocentric pattern: agent type mattered most when positive moral behavior no longer provided an independent basis for protection.

The observed interaction is consistent with the hierarchy‐of‐being perspective on moral status (Brandt and Reyna 2011). When agents had previously behaved morally, their positive behavior may have provided a sufficient basis for protection, making agent type less salient in children's appropriateness ratings. By contrast, when the saved agents had behaved immorally, positive behavioral information no longer provided an independent reason for protection, and children may have relied more strongly on agent type as an additional evaluative cue. Under this interpretation, the higher appropriateness ratings for saving immoral humans than immoral robots suggest that children may grant humans a baseline level of moral consideration even when they have behaved negatively.

This conditional anthropocentric pattern may be related to children's assumptions about sentience, biological vulnerability, and intrinsic moral standing (Decety and Cowell 2017; Perry 2024). Developmental work suggests that children often distinguish biological beings from artifacts and may view humans as possessing richer experiential capacities than robots (Gelman 2003; Sommer et al. 2019). Thus, an immoral human may still be perceived as a vulnerable being capable of suffering, whereas an immoral robot may be evaluated more as an artificial agent whose protection carries weaker moral weight. However, because the present study did not directly measure children's beliefs about sentience, pain, repairability, or consciousness, this interpretation should be treated as theoretically plausible rather than conclusive. Future research should directly assess these underlying attributions to clarify why human–robot differences emerge selectively when saved agents have behaved immorally.

## 4 General Discussion

The present research investigated how 5‐ to 6‐year‐old children integrate prior behavior and agent type (human vs. robot) when making normative judgments. By comparing the conventional domain (Study 1) and the moral domain (Study 2), the findings suggest a domain‐related dissociation between binary choices and explicit appropriateness ratings. Across both studies, children's choices were strongly organized by the behavioral valence of the sacrificial and saved targets. The repeated‐measures ANOVAs also suggested greater intervention in human than in robot scenarios, although these agent‐related choice effects were sensitive to model specification. In contrast, appropriateness ratings showed a clearer domain‐related pattern. In Study 1, children evaluated human and robot scenarios comparably, consistent with a relatively agent‐general application of conventional standards. In Study 2, however, children rated saving an immoral human as more appropriate than saving an immoral robot, whereas ratings did not differ when the saved agents were moral. Although the direct cross‐study interaction did not reach the conventional significance threshold, the pattern is directionally consistent with the proposal that children's evaluations of robots and humans vary across normative domains.

This pattern helps clarify the boundary conditions of children's social categorization of artificial agents. The conventional‐domain findings are consistent with an integration of CASA framework and social domain theory (Nass and Moon 2000; Smetana 2013; Turiel 1983, 2008). CASA explains why children may treat robots as socially relevant participants, whereas social domain theory specifies the criteria children use to evaluate conduct within a given normative domain. Conventional norms function as coordination rules that organize social interaction and are typically understood as context‐dependent and authority‐contingent (Smetana 2013; Turiel 2008). Once a robot is treated as a participant in such interactions, children may focus primarily on observable rule compliance rather than ontological category, a pattern consistent with prior work on children's application of social and fairness norms to robots (Ayalon et al. 2023; Severson and Lemm 2016). In this sense, robots can be evaluated as functional social partners in conventional contexts. The moral‐domain findings, by contrast, suggest a boundary to this generalization. Moral judgments are more closely tied to welfare, harm, vulnerability, and perceived sentience (Caviola et al. 2022; Cushman 2015; Gray et al. 2007). When the evaluation concerns morally consequential protection, children may give greater weight to the presumed biological and experiential capacities of humans than to the functional agency of robots.

At the same time, the present data do not uniquely identify the mechanism underlying this anthropocentric pattern. An essentialist interpretation is plausible: children may treat humans as natural kinds with biological vulnerability, while viewing robots as artifacts with weaker claims to moral protection. However, other explanations remain possible. Children may be more familiar with human suffering, may perceive humans as less repairable than robots, or may attribute richer mental and emotional experience to humans. These possibilities are consistent with work on psychological essentialism, mind perception, and children's judgments of the moral worth of different entities (Gelman 2003; Gray et al. 2007; Sommer et al. 2019; Wilks et al. 2020). Because children's beliefs about sentience, pain, consciousness, and repairability were not directly measured, the claim that humans were granted greater moral standing should be understood as a theoretically grounded interpretation rather than a direct empirical conclusion.

The dissociation between binary choices and appropriateness ratings is also compatible with a dual‐process‐informed distinction between categorical action selection and graded explicit appraisal (Cushman 2013; Greene 2007), but it does not directly identify separate cognitive systems. In the present paradigm, binary choices required children to make a categorical response within a fixed task structure, whereas appropriateness ratings allowed a more graded evaluation after a choice had been made. Thus, the two measures may have encouraged different weighting of target valence, agent type, and normative domain. In Study 1, graded evaluations primarily tracked rule compliance and did not differ significantly between human and robot scenarios. In Study 2, the scenario‐agent difference in ratings depended on the saved agents' prior moral behavior: saving an immoral human was rated as more appropriate than saving an immoral robot, whereas ratings did not differ when the saved agents were moral. Because both responses were self‐paced, processing time and cognitive load were not manipulated, and ratings always followed choices, this interpretation should be regarded as functional rather than process‐pure. Response granularity, post‐choice evaluation, and sequential consistency may also have contributed to the observed pattern.

## 5 Limitations and Implications for Further Research

Several limitations should be considered. First, the use of static visual stimuli may have reduced the perceived agency, responsiveness, or experiential capacity of the robot agents, thereby magnifying human–robot differences in moral evaluations. Future studies should use dynamic or interactive robot paradigms to examine whether embodiment and contingent interaction moderate these effects. Second, the narrow age range of 5‐ to 6‐year‐olds limits developmental generalizability. Because children's concepts of biology, mind, and artifact status change with age, longitudinal or broader cross‐sectional designs are needed to clarify how the observed pattern develops. Third, the sample was drawn from a single urban Chinese context, and children's prior experience with robots was not measured. Cultural norms and robot familiarity may influence whether children treat robots as social participants, artifacts, or members of a relevant social category. Fourth, the absence of animal comparison conditions limits our ability to distinguish a specifically human‐centered preference from a broader preference for biological or sentient entities. Including animals in future studies would provide a stronger test of the hierarchy‐of‐being interpretation. In addition, although the shared high‐stakes road‐accident task improved comparability across domains, it also reduced ecological validity, especially in the conventional domain, where rule violations are typically minor and do not involve life‐or‐death consequences. Finally, several choice conditions showed extreme ceiling or floor effects, which constrained variance and made the behavioral agent‐type effects sensitive to model specification. Future research should use tasks with more varied difficulty levels and richer response distributions to test the robustness of these effects.

## 6 Conclusions

The present study demonstrates that preschoolers' behavioral decisions generally favored humans and agents with positive prior behavior across both conventional and moral domains. However, explicit evaluations exhibited domain specificity. In the conventional domain, children evaluated decisions involving humans and robots comparably, suggesting that conventional norms may be applied in a relatively agent‐general manner when observable rule compliance is salient. In the moral domain, children showed a conditional anthropocentric pattern: saving an immoral human was rated as more appropriate than saving an immoral robot, whereas ratings did not differ when the saved agents were moral. These findings suggest that while preschoolers may treat robots as procedural social actors in conventional contexts, they may grant humans greater presumed moral standing in morally consequential contexts. Nevertheless, because the direct cross‐study interaction was not statistically significant and children's beliefs about sentience or suffering were not directly measured, the domain contrast should be interpreted as suggestive rather than conclusive.

## Funding

This work was supported by the Youth Fund for Humanities and Social Science of the Ministry of Education of China (24YJC880011).

## Conflicts of Interest

The authors declare no conflicts of interest.

## Data Availability

The data that support the findings of this study are available from the corresponding author upon reasonable request.
